# Examining Clinician Offer of Video Telehealth at Veterans Health Administration: A National Survey

**DOI:** 10.1093/geroni/igaf122.2800

**Published:** 2025-12-31

**Authors:** Megan Gately, Steven Shirk, Emily Metcalf, Dylan Waller, Emma Quach, Lauren Moo

**Affiliations:** VA Bedford Health Care System, Bedford, Massachusetts, United States; VA Bedford Health Care System, Bedford, Massachusetts, United States; VA Bedford Health Care System, Bedford, Massachusetts, United States; Center to Improve Veteran Involvement in Care (CIVIC), Portland, Oregon, United States; VA Bedford Health Care System, Bedford, Massachusetts, United States; VA Bedford Healthcare System, Bedford, Massachusetts, United States

## Abstract

Though older adults have historically utilized technology less than younger adults, their use of telehealth greatly expanded in recent years. Despite these gains, a telehealth Digital Divide has led to discrepancies in use of telehealth with older adults and others with complex medical needs, creating a persistent barrier to healthcare. While evidence for how clinicians decide which patients to offer video telehealth to is generally lacking, age is frequently cited by clinicians as a barrier, suggesting an ageist attitude towards older adults. This study will present findings from a national survey of clinicians at Veterans Health Administration (VHA), the largest integrated healthcare system in the United States. The survey, which is approved for launch in March 2025, will be sent to over 297,000 inter-professional VHA clinicians, including physicians, nursing and rehabilitation professionals, psychologists, and social workers. Survey items include factors influencing clinician offer of video, such as patient factors (age, comorbidity, and caregiver availability) and organizational influences (leadership buy-in, telehealth metrics, availability of administrative/technical support), as well as clinician attitudes towards patient fitness for telehealth more broadly. Building on this first ever national survey examining factors influencing clinician utilization of video telehealth, this study aims to promote clinicians’ successful integration of telehealth with older patients, thereby increasing patient access to care.

